# Supplementary material for: Identification of photoperiod-induced specific miRNAs in the adrenal glands of Sunite sheep (Ovis aries)
Source: Front Vet Sci. 2022 Jul 22;9:888207. doi: 10.3389/fvets.2022.888207 (PMC9354845; doi:10.3389/fvets.2022.888207)
Supplement: Supplementary file 1 [file Data_Sheet_1.ZIP › Supplementary file/miRDeep2 file.zip!/miRDeep2 file/Novel_Structure/SP42A3/Novel_29.pdf]

Provisional ID	:	NC_019484.2_32711
Score total	:	106.9
Score for star read(s)	:	3.9
Score for read counts	:	99
Score for mfe	:	2.4
Score for randfold	:	1.6
Score for cons. seed	:	
Total read count	:	206
Mature read count	:	203
Loop read count	:	0
Star read count	:	3

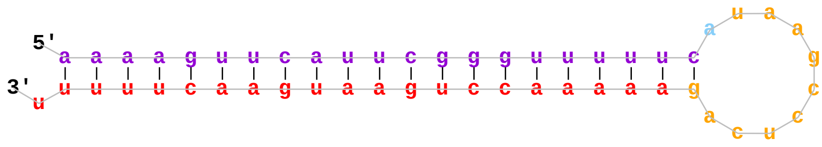

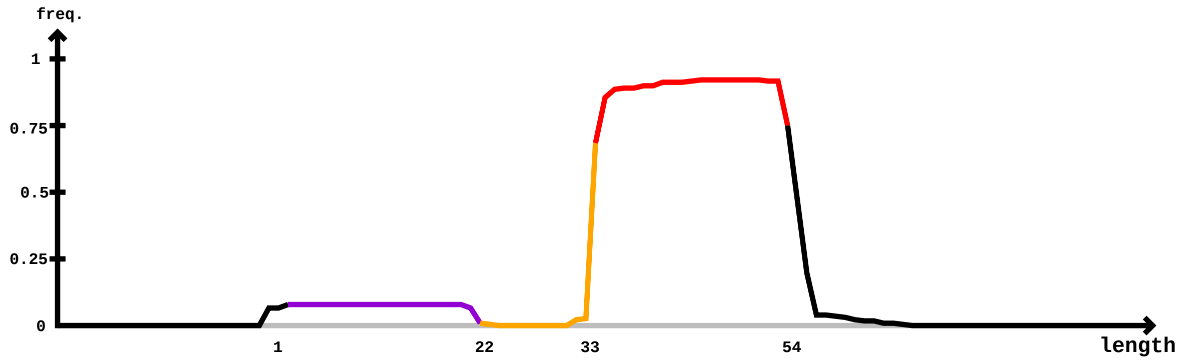

Star Mature

```
5'- cuaucauauauuggguuggcugaaaaguucauucggguuuuucauaagccucagaaaaaccugaaugaacuuuuuggccaacccaauauuaucuaacuaucccaguggaguc   -3'   obs
    cuaucauauauuggguuggcugaaaaguucauucggguuuuucauaagccucagaaaaaccugaaugaacuuuuuggccaacccaauauuaucuaacuaucccaguggaguc         exp
    .......((((((((((((((((((((((((((((((((((((..........))))))))))))))))))))))))))))))))))))..........(((....)))...   reads   mm   sample
    ....................ugaaaaguucauucggguuuu.......................................................................   3       0    seq
    ....................ugaaaaguucauucggguuuuu......................................................................   12      0    seq
    ......................aaaaguucauucggguuuuu......................................................................   1       0    seq
    ......................aaaaguucauuAggguuuuuc.....................................................................   2       1    seq
    ....................................................agaaaaaUcugaaugaacuuuuu.....................................   5       1    seq
    .....................................................gaaaaCccugaaugaacuuuuu.....................................   1       1    seq
    ......................................................aaaaaUcugaaugaacuu........................................   1       1    seq
    ......................................................aaaaaccugaaGgaacuuuu......................................   4       1    seq
    ......................................................aaaaaccugaaCgaacuuuu......................................   4       1    seq
    ......................................................aaaaaccAgaaugaacuuuuu.....................................   1       1    seq
    ......................................................aaaaaUcugaaugaacuuuu......................................   24      1    seq
    ......................................................aaaaaccugaaugaacuuuu......................................   3       0    seq
    ......................................................aaaaaccugaaGgaacuuuuu.....................................   8       1    seq
    ......................................................aaaaaccAgaaugaacuuuuu.....................................   2       1    seq
    ......................................................aaaaaUcugaaugaacuuuuu.....................................   69      1    seq
    ......................................................aaaaaccugaaugaacuuGuug....................................   1       1    seq
    ......................................................aaaaaccugaaugaacuuuuug....................................   22      0    seq
    ......................................................aaaaacUugaaugaacuuuuug....................................   1       1    seq
    ......................................................aaaaacAugaaugaacuuuuug....................................   1       1    seq
    ......................................................aaaCaccugaaugaacuuuuug....................................   2       1    seq
    ......................................................aaaaaccugaaugaacuuuuugU...................................   3       1    seq
    ......................................................aaaaaccugaaugaacuuuuugC...................................   1       1    seq
    ......................................................aaaaaccugaaugaacuuuuugA...................................   3       1    seq
    .......................................................aaaaccugaaugaacuuuu......................................   1       0    seq
    .......................................................aaaaccugaaugaacuuuC......................................   1       1    seq
    .......................................................aaaaccAgaaugaacuuuuu.....................................   3       1    seq
    .......................................................aaaaccugaaugaacuuuuug....................................   9       0    seq
    .......................................................aaaaccugaaugaacCuuuug....................................   1       1    seq
    .......................................................aaaaccugaaugaacuuuuugA...................................   11      1    seq
    .......................................................aaaaccugaaugaacuuuuugU...................................   10      1    seq
    .......................................................aaaaccugaauAaacuuuuugg...................................   1       1    seq
    .......................................................aGaaccugaaugaacuuuuugg...................................   1       1    seq
    .......................................................aaaGccugaaugaacuuuuugg...................................   1       1    seq
```

```
                      Star                            Mature
cuaucauauauuggguuggcugaaaaguucauucggguuuuucauaagccucagaaaaaccugaaugaacuuuuuggccaacccaauauuaucuaacuaucccaguggaguc
........................................................aaaccAgaaugaacuuuuug....................................    1    1    seq
........................................................aaGccugaaugaacuuuuugg...................................    1    1    seq
........................................................aaaccugaaugaacuuuuugg...................................    3    0    seq
........................................................aaaccugaaugaacuuuuuggcU.................................    1    1    seq
........................................................aaacUugaaugaacuuuuuggcca................................    1    1    seq
.........................................................aaccugGaugaacuuuuugg...................................    1    1    seq
...........................................................ccugaaugaacuuuuuggccaa...............................    2    0    seq
.............................................................ugaaugaacuuuuuggccaac..............................    1    0    seq
.............................................................ugaaugaacuuuuuggccaaccA............................    1    1    seq
.............................................................ugaaugaacuuuuuggccaaccU............................    1    1    seq
................................................................augaacuuuuuggccaacccaa..........................    1    0    seq
.................................................................ugaaUuuuuuggccaacccaau.........................    1    1    seq
```
